# Supplementary material for: Adolescent obesity induces sex-specific alterations of action control
Source: Neuropsychopharmacology. 2026 Apr 23;51(9):1699–708. doi: 10.1038/s41386-026-02419-w (PMC13389461; doi:10.1038/s41386-026-02419-w)
Supplement: Supplementary file 1 — Supplemental Figures 1–5 [file 41386_2026_2419_MOESM1_ESM.docx]

Adolescent obesity induces sex-specific alterations of action control

**Authors:** Diptendu Mukherjee^1,2#^, Solenne Rougeux^1,2#^, Robert T. West^1,2^, Ahlima Roumane^1,2^, Kate Z. Peters^1,3^, Fabien Naneix^1,2^*

**Supplemental Figures and Tables**

**
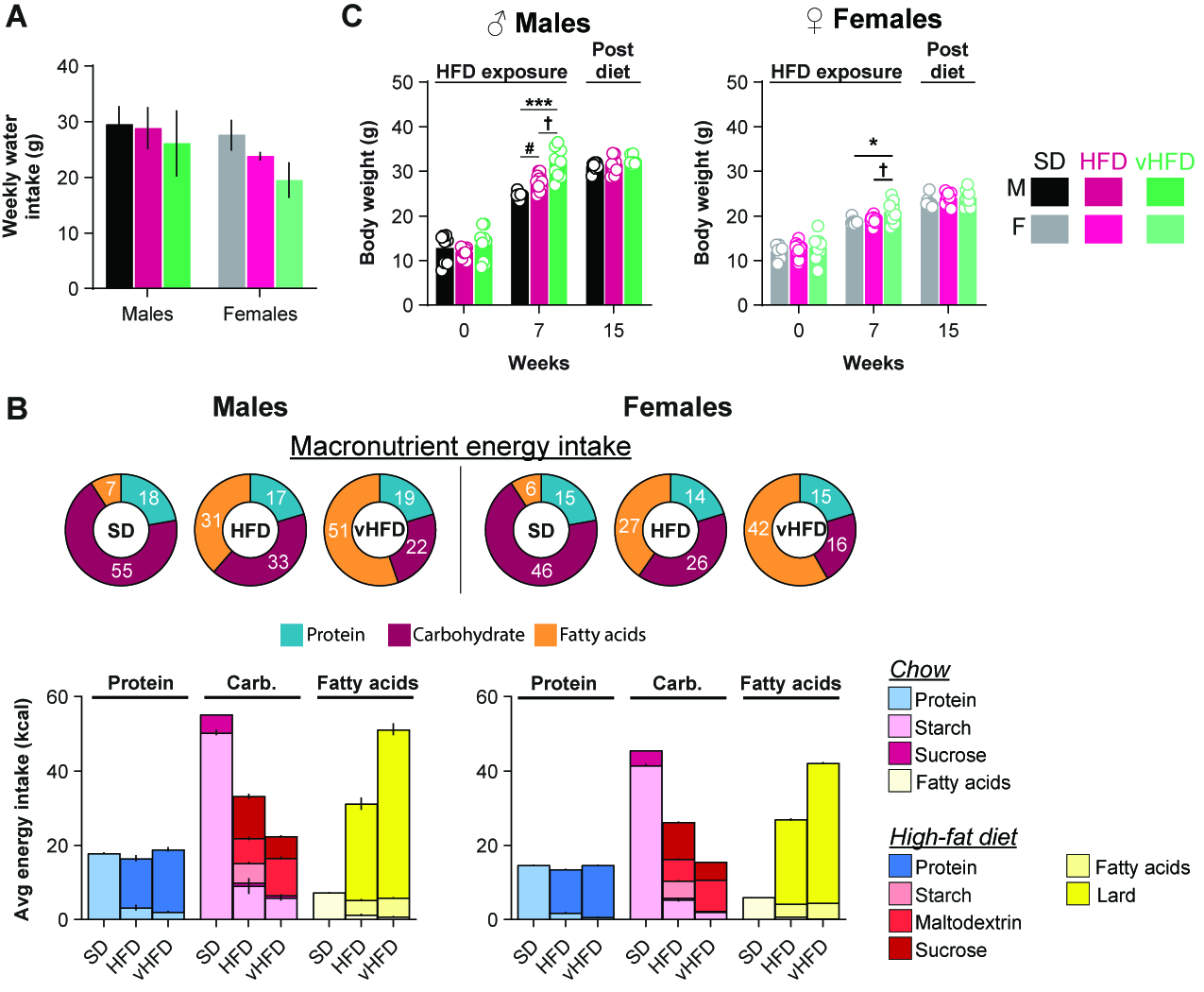
**

**Supplemental Figure 1. A.** Weekly water intake (g, estimated from cage consumption) is similar across all groups. **B.** Macronutrient breakdown for average weekly energy intake (in kcal) during diet exposure. Nutrient type is specifically color coded depending on the food source (chow or high-fat diet). **C.** Body weight at the start (0; Males: F_(2, 25)_ = 0.9, p = 0.4, η^2^ = 0.07 / Females: F_(2, 26)_ = 0.4, p = 0.7, η^2^ = 0.03), the end of diet exposure (5 weeks; Males: F_(2 ,25)_ = 22.6, p < 0.001, η^2^ = 0.64 / Females: F_(2 ,26)_ = 5.3, p = 0.01, η^2^ = 0.29) and at the end of behavioral testing (15 weeks; Males: F_(2, 22)_ = 1.5, p = 0.3, η^2^ = 0.12 / Females: F^(2, 24)^ = 1.3, p = 0.3, η^2^ = 0.10). SD, 8 M/8F (black/gray respectively); HFD, 11M/12F (dark and light pink); vHFD, 9M/9F (dark and light green). Data are presented as mean ± SEM with individual values (open circles). * p < 0.05, *** p < 0.001 SD vs vHFD; # p < 0.05 SD vs HFD; † p < 0.05 HFD vs vHFD (one-way or two-way ANOVA followed by Bonferroni’s *post hoc* tests). Full statistical reporting is provided in **Supplemental Table 3**.

**
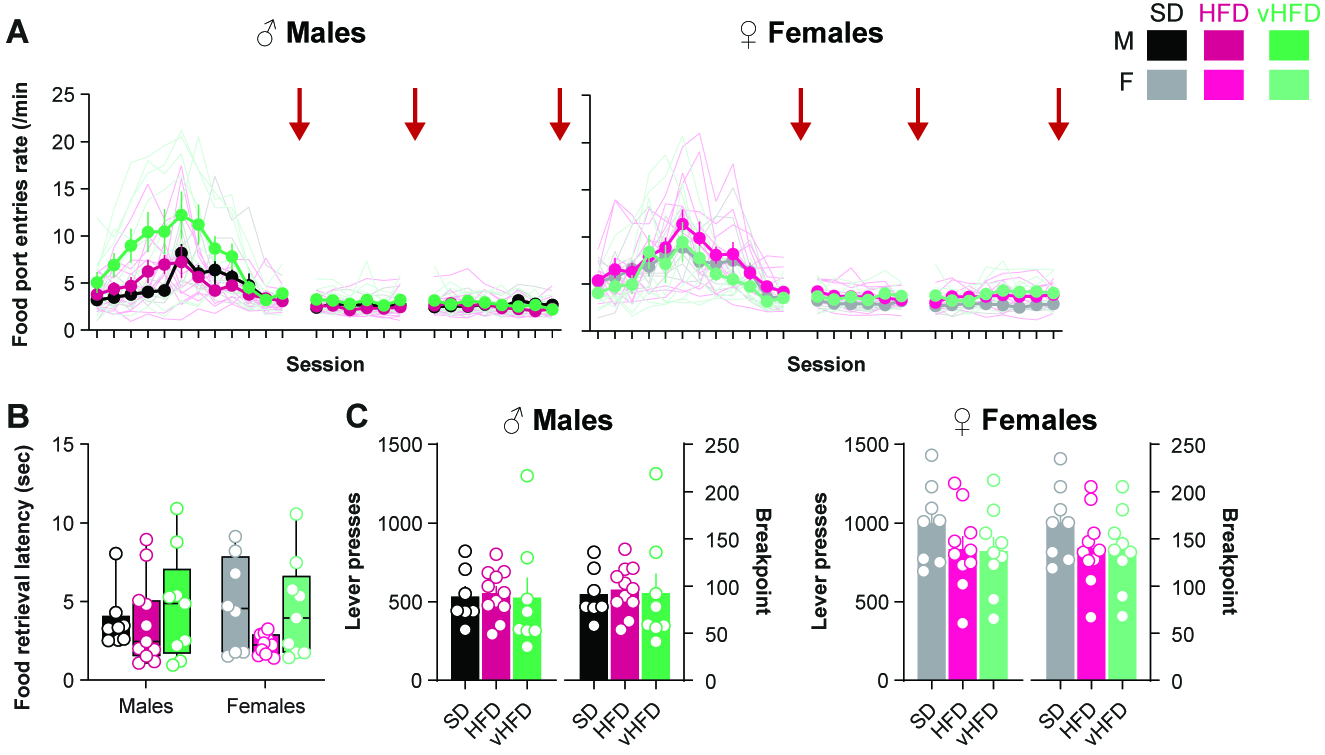
**

**Supplemental Figure 2. A.** Food port entries response rate across initial training. Red arrows indicate the timing of the outcome devaluation tests. **B.** Average food retrieval latency during initial instrumental training. **C.** Progressive ratio task. Average total lever presses and breakpoint for male and female groups. SD, 7-9M/8-9F (black/gray respectively); HFD, 11M/10-11F (dark and light pink); vHFD, 8-9M/9F (dark and light green). Data are presented as mean ± SEM (bars or closed circles) with individual values (open circles or thin lines). Full statistical reporting is provided in **Supplemental Table 3**.

**
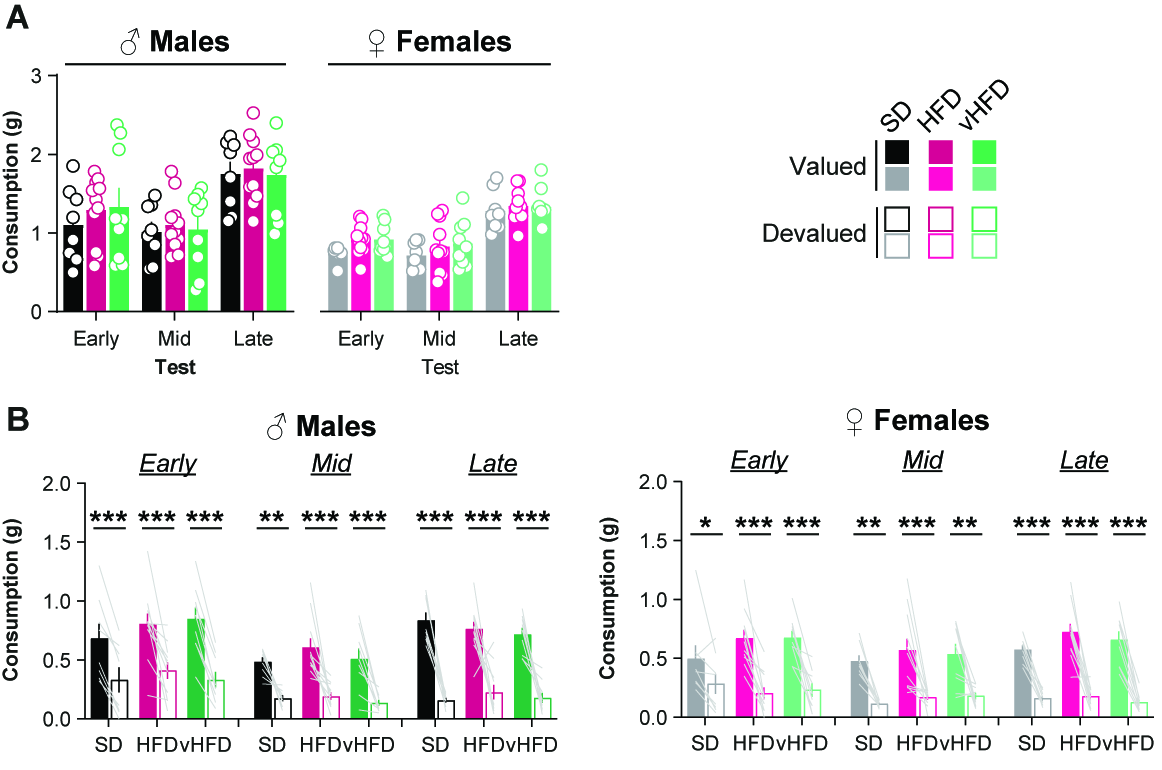
**

**Supplemental Figure 3. A.** Food consumption (g) during the satiety phase of early, mid and late outcome devaluation tests. **B.** Food consumption (g) the choice consumption tests after each outcome devaluation test. SD, 8M/8F (black/gray respectively); HFD, 11M/11F (dark and light pink); vHFD, 9M/9F (dark and light green). Data are presented as mean ± SEM (bars or closed circles) with individual values (open circles or thin lines). *, **, *** p < 0.05, 0.01 and 0.001 respectively Devaluation effect (two-way ANOVA followed by Bonferroni’s *post hoc* tests). Full statistical reporting is provided in **Supplemental Table 3**.

**
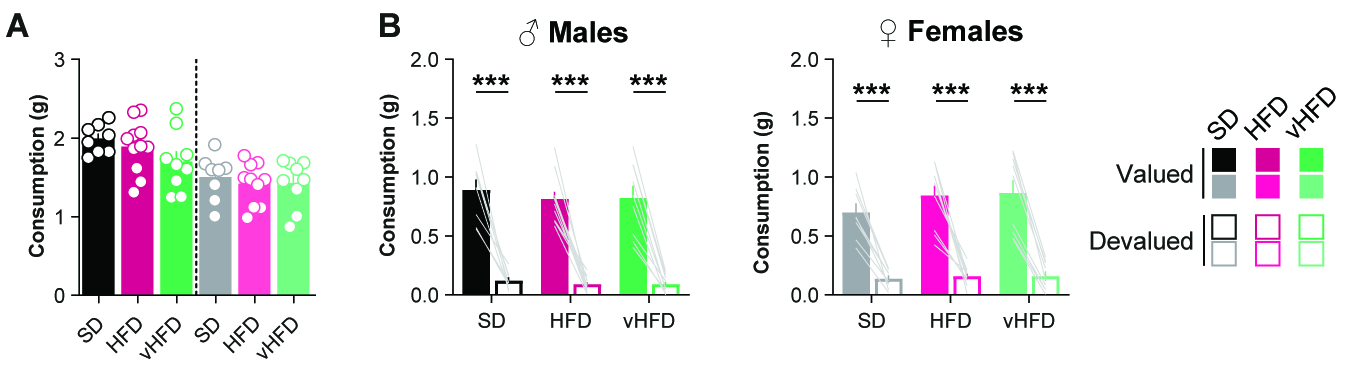
**

**Supplemental Figure 4. A.** Food consumption (g) during the outcome devaluation tests after reversal learning. **B.** Food consumption (g) the choice consumption tests after the outcome devaluation test. SD, 8M/8F (black/gray respectively); HFD, 11M/10F (dark and light pink); vHFD, 9M/9F (dark and light green). Data are presented as mean ± SEM (bars or closed circles) with individual values (open circles or thin lines). *, **, *** p < 0.05, 0.01 and 0.001 respectively Devaluation effect (two-way ANOVA followed by Bonferroni’s *post hoc* tests). Full statistical reporting is provided in **Supplemental Table 3**.

**
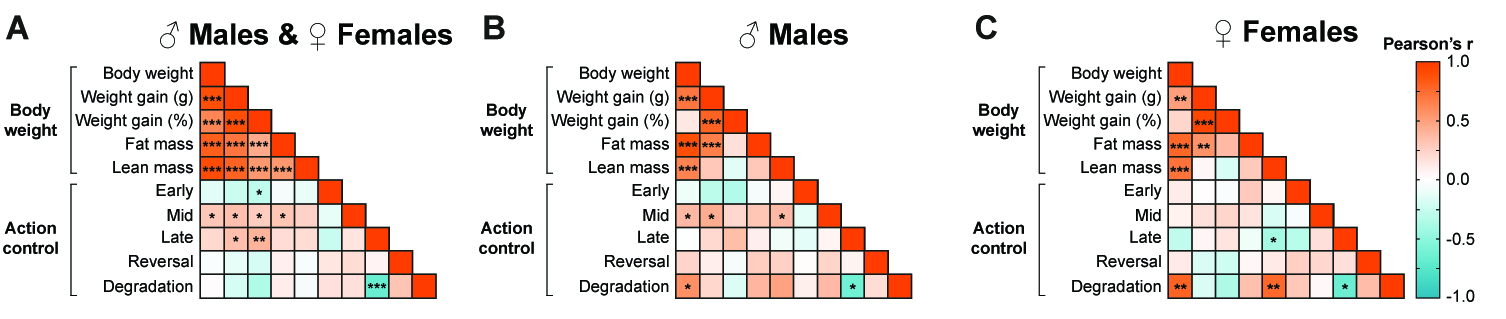
**

**Supplemental Figure 5.** Correlation matrices between body weight measures and action control performance. Heatmaps showing the relationship (Pearson’s r) between body weight at the end of diet exposure, weight gain (g), weight gain (% starting body weight), fat and lean mass (g), Outcome Devaluation indexes (Early, Mid, Late, Reversal; Response rate Devalued/Valued+Devalued) and Contingency Degradation index (Response rate Degraded/Degraded+Baseline) for all mice (A), Males (B) and Females (C). *, **, *** p < 0.05, 0.01 and 0.001 respectively. Full statistical reporting is provided in **Supplemental Table 4**.
